# Complete elimination of hyperleukocytosis risk in AML through early high-quality disease remission

**DOI:** 10.3389/fonc.2024.1412583

**Published:** 2024-06-13

**Authors:** Guoqing Lyu, Wenting Lyu

**Affiliations:** ^1^ Department of Hematology, the First Affiliated Hospital of Xinxiang Medical University, Xinxiang, China; ^2^ Life Science Center, the First Affiliated Hospital of Xinxiang Medical University, Xinxiang, China; ^3^ Key Laboratory for Leukemia Molecular Diagnosis and Treatment in Xinxiang, the First Affiliated Hospital of Xinxiang Medical University, Xinxiang, China; ^4^ Key Laboratory for Lymphoma Molecular Diagnosis and Treatment in Xinxiang, the First Affiliated Hospital of Xinxiang Medical University, Xinxiang, China; ^5^ School of Medicine, Pingdingshan University, Pingdingshan, China

**Keywords:** hyperleukocytosis (HL), leukemia, acute myeloid leukemia (AML), cytoreduction, dynamic assessment

## Abstract

**Background:**

Acute myeloid leukemia (AML) with hyperleukocytosis (HL) is a severe medical emergency associated with high mortality rates and poor prognosis. Prompt and urgent treatment is crucial to address this medical emergency. This study aims to elucidate appropriate diagnostic thresholds for HL and investigate underlying mechanisms and potential targeted therapies.

**Methods:**

X-tile software was employed to analyze white blood cell (WBC) count thresholds in AML patients using data from TCGA and TARGET AML databases. METASCAPE and Gene Set Enrichment Analysis (GSEA) were conducted to explore the molecular mechanisms underlying HL in AML. Potential molecular targeted drugs were identified using the CELLMINER platform.

**Results:**

Analysis revealed that a WBC count threshold of 75×10^9^/L, rather than the conventional 100×10^9^/L, is more appropriate for diagnosing HL in adult AML patients. This revised threshold could aid clinicians in identifying a greater number of patients requiring immediate intervention. Significant correlations were observed between HL and specific mutations, including NPM1, FLT3, and DNMT3A. For pediatric AML patients, the HL threshold was determined to be 165×10^9^/L. Achieving complete remission (CR) or deeper levels of remission significantly reduces the risks associated with HL. The reduction in risk can lead to survival outcomes for HL patients that are comparable to those of non-hyperleukocytosis patients. Differential gene expression analysis indicated that downregulation of cell adhesion molecules is implicated in HL pathogenesis. Potential targeted therapies for AML with HL include Bcl2 inhibitors and histone deacetylase inhibitors. Clinical observations demonstrated that the addition of Bcl2 inhibitors, such as Venetoclax, to standard therapy results in a rapid reduction in WBC counts, thereby reducing tumor burden and providing prompt symptom relief. Combining these targeted drugs with conventional therapies appears promising in mitigating risks associated with HL.

**Conclusions:**

Lower diagnostic thresholds for HL in AML, identifies critical genetic correlations, and highlights effective molecular targeted therapies. Proactive early treatment is crucial for achieving deep remission and reducing HL risk. Future therapeutic strategies should consider integrating molecular targeted drugs with conventional therapies to improve outcomes for patients facing this high-risk hematological emergency.

## 1 Introduction

AML is a complex and heterogeneous hematological malignancy characterized by the abnormal proliferation of myeloid progenitor cells (1). One notable laboratory abnormality observed in AML is HL, which refers to an elevated white blood cell count. Although HL is commonly defined as a white blood cell count exceeding 100 ×10^9^/L, it should be noted that even levels below this threshold can lead to complications associated with HL (2–5).

HL represents a medical emergency frequently observed in patients with AML. It presents with rapid disease progression and significantly increased mortality rates, resulting in poor prognosis. The specific molecular mechanisms underlying HL in AML remain incompletely understood (2, 5–10).

Therefore, the primary objective of this study is to explore and define the optimal threshold for diagnosing HL and elucidate the potential molecular mechanisms of HL in order to gain deeper insights into this critical aspect of AML pathogenesis. By better understanding the underlying molecular mechanisms, we hope to identify promising therapeutic targets that can be exploited for the development of more effective treatment strategies aimed at improving outcomes for patients with AML and HL.

## 2 Materials and methods

### Clinical case

A newly diagnosed patient with AML and HL exhibited poor response to hydroxyurea treatment. However, combination therapy involving the administration of the Bcl2 inhibitor venetoclax showed significant improvement. This study was approved by the Human Research Ethics Committee of First Affiliated Hospital of Xinxiang Medical University (NO.2020156).

### TCGA analysis

Gene expression data and clinical data of adult AML patients were downloaded from the TCGA website.

### TARGET analysis

Clinical data of children AML patients were downloaded from the TARGET website.

### X-tile analysis

The X-tile analysis method was employed in this study to categorize data into distinct groups based on specified cut-off values. This statistical technique is commonly used for determining optimal thresholds or breakpoints within a data set. X-tile analysis was employed to determine the optimal threshold for WBC count in patients with AML with HL.

### Metascape analysis

Gene ontology (GO) term enrichment analysis and pathway analysis using Metascape (https://metascape.org/)to explore the functional characteristics and underlying pathways associated with differentially expressed genes in patients with AML with HL (11).

### Gene set enrichment analysis

Gene set enrichment analysis was performed by using GSEA version 4.0.3 software to analyze the gene set characteristics in patients diagnosed with AML and HL.

### Statistical processing

All statistical analyses were performed by R software 4.3.2, SPSS software 26.0 and GraphPad Prism software 9.0. The measurement data were expressed as mean ± standard deviation, and t-test was used for comparison. Descriptive statistics were used to summarize the clinical characteristics of the patients. Datasets were described by median and/or range. Between group comparisons of numerical and categorical data were performed by the Mann-Whitney *U* test and the chi-square test, respectively. All tests were two-tailed. Statistical significance was defined as *P*<0.05.

## 3 Results

### The ideal threshold for WBC count in adult patients diagnosed with AML with HL

**Figure 1:**
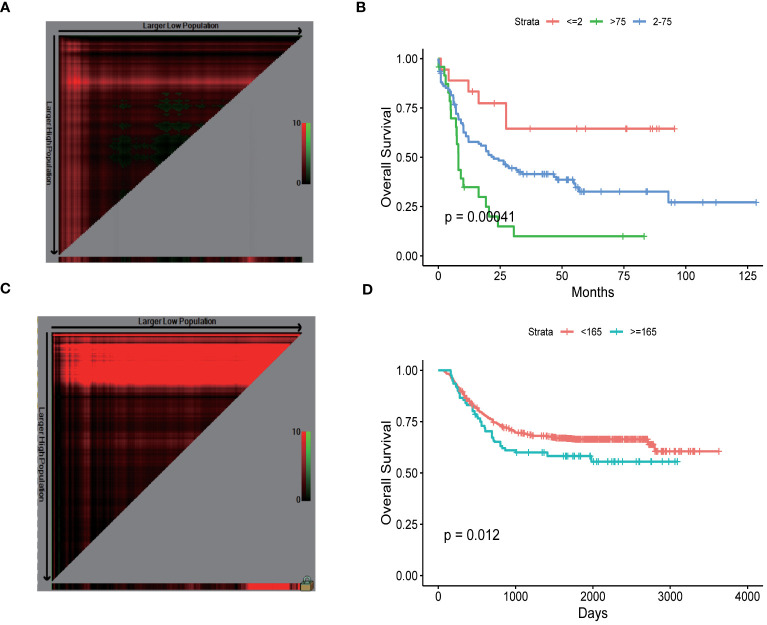
The ideal threshold for WBC count in patients diagnosed with adult AML with HL **(A, B)**. adult AML, **(C, D)**. pediatric AML.

X-tile software was utilized to determine the ideal threshold for WBC count in adult patients diagnosed with AML with HL. The findings revealed that AML patients could be stratified into three prognostic groups based on their leukocyte counts: <2, 2.20–72.10, and >75*10^9^/L. These distinct groupings demonstrated significant differences in terms of prognosis among the AML patients. We observed that lower leukocyte counts were associated with more favorable prognoses, whereas higher leukocyte counts indicated poorer prognoses in AML patients. This can aid clinicians in assessing disease severity and making well-informed decisions regarding treatment strategies. The traditional threshold of 100 ×10^9^/L has been used as a marker for identifying patients at super high risk and requiring urgent further investigation. According to our research findings, it is feasible to lower the threshold for HL from 100 ×10^9^/L to 75*10^9^/L. This adjustment in the diagnostic criteria may have significant implications in clinical practice. Our study suggests that lowering this threshold to 75*10^9^/L can still effectively capture individuals who may benefit from timely intervention or closer monitoring (**Figures 1A, B**).

### Clinical characteristics of adult patients diagnosed with AML with HL

Data from the TCGA adult AML study were analyzed to identify patients with HL. Using the initial threshold of a WBC count exceeding 100*10^9^/L, it was estimated that approximately 10% of adult AML patients exhibited HL. However, upon lowering the threshold to 75 ×10^9^/L, approximately 18% of patients were found to meet the criteria for HL. This adjustment resulted in a nearly twofold increase in the number of patients diagnosed with HL. Lowering the threshold for HL allows for the identification of a larger subset of adult AML patients with HL. This adjustment in diagnostic criteria has important implications for clinical practice, as it enables the identification and subsequent treatment of a greater number of super high-risk patients. The availability of more aggressive and effective treatment options for these patients may lead to improved outcomes and survival rates.

Data from adult AML patients were analyzed using a cutoff value of 75 ×10^9^/L for WBC count to distinguish between patients with HL and those without HL. The association between HL and various clinical characteristics was investigated. The findings of our study demonstrated that HL did not show any significant correlation with gender, age, FAB, CR(%), early mortality, different layers of cytogenetic risk and molecular risk classifications. However, our analysis revealed that HL showed significant associations with total WBC count, peripheral Blast ratio and bone marrow Blast ratio. In patients with HL, there is a significant increase in the number of leukemic cells in the bone marrow and peripheral blood, which is notably higher than in non-HL patients. Furthermore, our study identified significant correlations between HL and specific genetic markers, NPM1,FLT3, DNMT3A, BCR-ABL1, HECW1 and GATA2. In patients with HL leukemia, over half of the patients exhibit FLT3 gene mutations, and mutations in DNMT3A and NPM1 genes are also present in nearly half of the cases. In contrast, in non-HL leukemia, the proportion of these gene mutations is only one-fourth (**Table 1**).

**Table 1:** Clinical characteristics of HL.

Characteristic	N	Overall, N = 180	AML		p-value
			HL, N = 34	Non-HL, N = 146	
Sex	180				0.9
F		82 (46%)	15 (44%)	67 (46%)	
M		98 (54%)	19 (56%)	79 (54%)	
FAB	180				0.7
M0		19 (11%)	3 (8.8%)	16 (11%)	
M1		46 (26%)	9 (26%)	37 (25%)	
M2		44 (24%)	6 (18%)	38 (26%)	
M4		41 (23%)	9 (26%)	32 (22%)	
M5		22 (12%)	6 (18%)	16 (11%)	
M6		3 (1.7%)	0 (0%)	3 (2.1%)	
M7		3 (1.7%)	0 (0%)	3 (2.1%)	
nc		2 (1.1%)	1 (2.9%)	1 (0.7%)	
Age	180	59 (45, 67)	57 (44, 64)	60 (45, 68)	0.3
WBC(×109/L, median, range)	180	19 (5, 54)	103 (90, 128)	12 (3, 34)	<0.001
BM.Blast%	180	72 (53, 85)	78 (66, 87)	70 (51, 84)	0.015
PB.Blast%	178	39 (8, 67)	71 (51, 87)	29 (6, 57)	<0.001
RISK.Cyto.	180				0.13
Good		19 (11%)	2 (5.9%)	17 (12%)	
Intermediate		113 (63%)	27 (79%)	86 (59%)	
N.D.		5 (2.8%)	1 (2.9%)	4 (2.7%)	
Poor		43 (24%)	4 (12%)	39 (27%)	
RISK.Molecular.	180				0.2
Good		19 (11%)	2 (5.9%)	17 (12%)	
Intermediate		106 (59%)	25 (74%)	81 (55%)	
N.D.		4 (2.2%)	1 (2.9%)	3 (2.1%)	
Poor		51 (28%)	6 (18%)	45 (31%)	
FLT3	180				<0.001
wt		130 (72%)	16 (47%)	114 (78%)	
mutation		50 (28%)	18 (53%)	32 (22%)	
NPM1	180				0.005
wt		126 (70%)	16 (47%)	110 (75%)	
mutation		54 (30%)	18 (53%)	36 (25%)	
DNMT3A	180				0.045
wt		129 (72%)	19 (56%)	110 (75%)	
mutation		51 (28%)	15 (44%)	36 (25%)	
TP53	180				>0.9
wt		164 (91%)	34 (100%)	130 (89%)	
mutation		16 (0.6%)	2 (0%)	16 (11%)	
HECW1	180				0.035
wt		178 (99%)	32 (94%)	146 (100%)	
mutation		2 (1%)	2 (6%)	0 (0%)	
GATA2	180				0.035
wt		178 (99%)	32 (94%)	146 (100%)	
mutation		2 (1%)	2 (6%)	0 (0%)	
BCR-ABL1	180				0.042
negative		177 (98%)	32 (94%)	145 (99%)	
BCR-ABL1		3 (1.7%)	2 (5.9%)	1 (0.7%)	
Complete remission(n, %)	161				0.9
CR1		150(83.3%)	29(85.3%)	121(82.9%)	
CR2		12(6.7%)	2(5.9%)	10(6.8%)	
Early mortality	25				0.8
1 month		21 (11.7%)	4 (11.8%)	17 (11.6%)	
2 month		25 (13.9%)	5 (14.7%)	20 (13.7%)	

### The correlation between HL and DNMT3A,NPM1,FLT3 mutations

**Figure 2:**
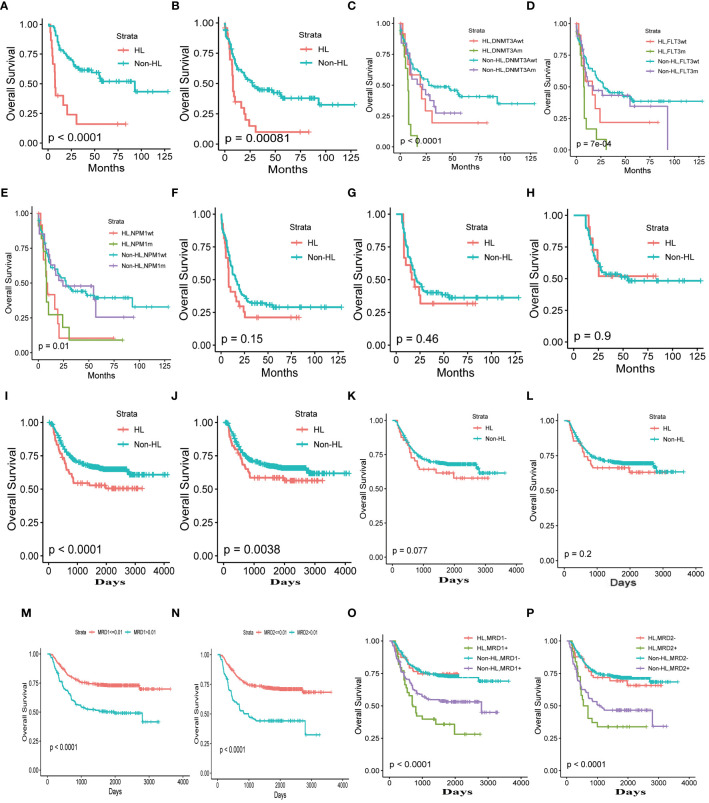
Clinical Characteristics of HL **(A)** Total adult AML. **(B)** Total adult AML excluding patients with acute promyelocytic leukemia. **(C–E)**. The correlation between HL and DNMT3A, NPM1, FLT3 mutations. **(F)** complete remission patients. **(G)**, The overall survival time exceeding 6 months, one year **(H)**. **(I)** Total pediatric patients with AML. **(J)** Total pediatric patients with AML excluding patients with acute promyelocytic leukemia. Complete remission after the first **(K)** and second courses of chemotherapy **(L)**. The thresholds for minimal residual disease after the first **(M)** and second cycles of chemotherapy **(N)**. Minimal residual disease after the first **(O)** and second cycles of chemotherapy **(P)**.

Using a WBC count threshold value of 75*10^9^/L to define HL, our analysis revealed a significantly worse overall survival in HL patients compared to non-HL (p<0.001). Even after excluding patients with acute promyelocytic leukemia, the analysis still showed a significant statistical overall survival difference between the two groups (p<0.001) (**Figures 2A, B**).

Our study revealed a correlation between HL and several gene mutations, including NPM1, FLT3, and DNMT3A. Patients with HL exhibited a more adverse prognosis, particularly when FLT3, and DNMT3A gene mutations coexisted with these mutations. Among the gene mutations, the prognosis was poorest for HL patients with DNMT3A (p<0.001)or FLT3 gene mutations (p<0.001). The NPM2 gene does not seem to have a significant impact on the prognosis of patients with AML with HL. (**Figures 2C–E**).

### Dynamic assessment of hyperleukocytosis risk

HL is a critical hematological emergency associated with high-risk leukemia. To address the dynamic assessment of hyperleukocytosis risk, we found that achieving complete remission or a deeper level of remission in patients with hyperleukocytosis significantly reduces the death risks. This suggests that the survival outcomes of patients with hyperleukocytosis can be improved to levels comparable to those without hyperleukocytosis, provided that remission is achieved (**Figure 2F**).

Moreover, the risk of hyperleukocytosis decreases as the patient’s survival time increases. As patient survival time increased, the risk of HL on prognosis was completely eliminated for patients with a survival time exceeding 6 months (p=0.48) (**Figure 2G**) and one year (p=0.9) (**Figure 2H**). Consequently, the survival rates of patients with hyperleukocytosis can approach those of patients without this condition over time.

### WBC counts exceeding 75 × 10^9^/L emerged as the only independent prognostic factor

In the univariate Cox regression analysis, our study identified that leukocyte counts exceeding both 75 ×10^9^/L and 100 × 10^9^/L were associated with poorer overall survival outcomes (HR 2.96, 95%CI: 1.99–5.04, p = 0.003; HR 1.76, 95%CI: 1.41–1.96, p = 0.02, respectively). However, in the multivariate Cox regression analysis, leukocyte counts greater than 75 ×10^9^/L emerged as the only independent prognostic factor for poor survival (HR 1.99, 95%CI: 1.52–3.53, p = 0.03) (**Table 2**).

**Table 2:** Univariate and multivariate analyses of OS in adult AML HL patients.

	Univariate analysis		Multivariate analysis	
Variables	HR(95%CI)	P value	HR(95%CI)	P value
Age	2.49(1.20–3.79)	0.001	1.13(1.01–2.07)	0.001
Sex	0.85(0.41–1.32)	0.6	1.09(0.49–1.31)	0.5
WBC(>=10×10^9^/L)	0.88(0.58–1.34)	0.6	1.15(0.65–1. 56)	0.8
WBC(>=50×10^9^/L)	1.01 (0.46–1.08)	0.13	1.06(0.69–3.79)	0.4
WBC(>=75×10^9^/L)	2.96(1.99–5.04)	0.003	1.99(1.52–3.53)	0.03
WBC(>=100×10^9^/L)	1.76(1.41–1.96)	0.02	1.24(0.84–1.69)	0.6
WBC(>=200×10^9^/L)	1.26(0.26–1.99)	0.5	1.53 (0.69–1.85)	0.6

These findings highlight the significance of leukocyte count thresholds in predicting patient outcomes. Specifically, while a leukocyte count above 100 ×10^9^/L is indicative of poor prognosis in a univariate context, a count exceeding 75 ×10^9^/L remains a robust independent prognostic marker when controlling for other variables. This underscores the need for vigilant leukocyte monitoring and potentially more aggressive therapeutic strategies for patients whose leukocyte counts surpass this critical threshold.

### The HL in pediatric AML patients

In pediatric patients with AML, we analyzed the impact of white blood cell (WBC) count on prognosis. Using the X-tile software, we sought to identify the optimal threshold of WBC count for predicting outcomes. It is important to note that the prognosis of pediatric AML patients is significantly better than that of adult AML patients. Therefore, the identified HL threshold for WBC count in pediatric AML patients was determined to be 165*10^9^/L. (**Figures 1C, D**).

Analyzing the impact of HL on prognosis of pediatric patients with AML using the optimal threshold of 165×10^9^, the overall survival of HL patients was significantly worse compared to non-HL patients (p<0.001). Even after excluding the influence of acute promyelocytic leukemia, the prognosis of HL patients remained inferior to that of non-HL patients. This finding is consistent with adult acute myeloid leukemia (p=0.0038) (**Figures 2I, J**).

### Complete elimination of HL risk in AML through early high-quality disease remission

Similar to HL in adult AML, the prognosis of HL patients in pediatric AML becomes similar to that of non-HL patients when complete remission is achieved after the first and second courses of chemotherapy ((p=0.077, p=0.2, respectively) (**Figures 2K, L**). Can the risk of HL be eliminated with deeper disease remission? We used X-tile software to analyze the optimal threshold of minimal residual disease in prognosis and found that the thresholds for minimal residual disease after the first and second cycles of chemotherapy were both 0.01 (**Figures 2M, N**). Analyzing HL using a threshold of 0.01 for minimal residual disease, we observed that the risk of HL is completely eliminated when the minimal residual disease is less than 0.01. However, when patients fail to achieve complete negativity for minimal residual disease, the risk of patients with HL is significantly higher than in patients without HL (**Figures 2O, P**).

### Differentially expressed genes associated with HL

A comprehensive differential gene analysis was conducted using the TCGA database to compare patients with HL to those non-HL. The aim of this analysis was to identify genes that are differentially expressed between these two groups and potentially associated with the pathogenesis of HL. A total of 844 differentially expressed genes were identified. Among these, 59 genes were found to be upregulated, while 785 genes showed downregulation in patients with HL compared to those non-HL. Several of the differentially expressed genes exhibited remarkable biological significance, including cell adhesion molecules such as OCLN, VCAM1, and CDH11. Cell adhesion molecules play crucial roles in mediating cellular interactions and are involved in various physiological processes such as immune response and inflammation. Their dysregulation can contribute to abnormal cell adhesion and migration, which may have implications for the development and progression of HL. Furthermore, cytokines such as CCL14, CXCL12 (also known as SDF-1), and IL33 were also among the differentially expressed genes identified in this analysis. Cytokines are signaling molecules that regulate immune responses by promoting communication between cells. Dysregulated cytokine expression has been implicated in various pathological conditions including inflammation-related disorders (**Table 3**).

**Table 3:** Differential Genes in HL.

GeneID	LOG_FC	P_VALUE
RNF17	20.92	0.019
RGPD3	5.61	0.021
ADRA1D	5.01	0.041
GOLGA6L5	3.97	0
NEGR1	3.92	0.041
IFNW1	3.81	0.043
WFIKKN2	3.81	0.017
APOC4	3.72	0.042
GBP6	3.71	0.038
OCLN	3.4	0.04
CLCN4	0.02	0.012
ID4	0.02	0.011
NTRK2	0.02	0.002
CCL14	0.02	0.024
CXCL12	0.01	0.019
CDH11	0.01	0.005
IL33	0.01	0.017
VCAM1	0.01	0.028
YAP1	0.01	0.016

### Potential molecular mechanisms of AML HL

**Figure 3:**
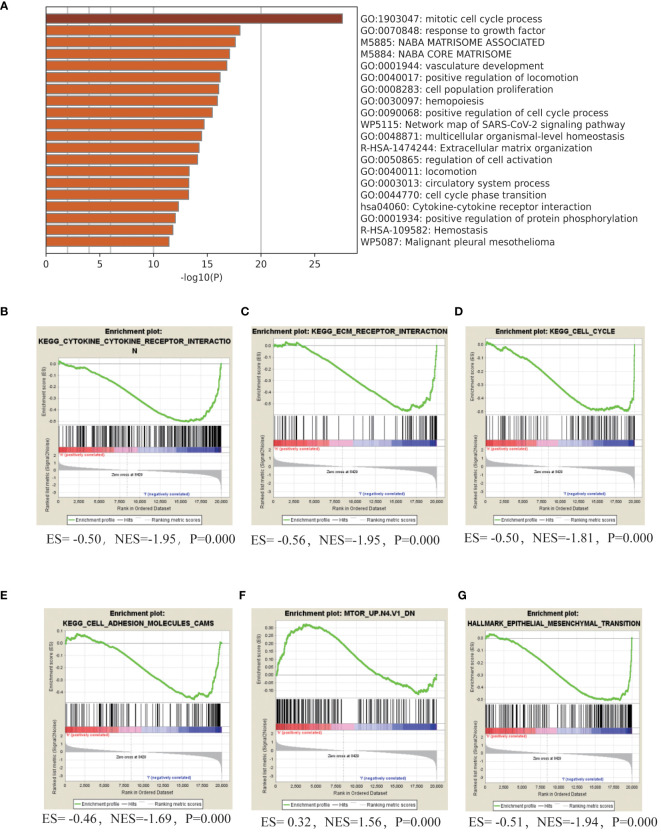
Molecular Mechanisms of HL in adult AML **(A)**. Gene ontology (GO) term enrichment analysis and pathway analysis using Metascape in adult patients with AML with HL. **(B–G)**. GSEA analyze the gene set characteristics in patients diagnosed with HL.

To understanding the intricate molecular processes contributing to HL in AML, METASCAPE analysis and Gene set enrichment analysis (GSEA) was conducted to explore the potential molecular mechanisms underlying HL in acute myeloid leukemia. The analysis unveiled several gene sets that exhibited a negative association with this condition, including cytokine-cytokine receptor interaction, extracellular matrix-receptor interaction, cell cycle regulation, DNA replication, cell adhesion molecules, cytokine signaling pathway, adhesion signaling pathway, and Epithelial Mesenchymal Transition (EMT). Furthermore, we identified a positively correlated gene set associated with the mammalian target of rapamycin (mTOR) signaling pathway in patients with AML HL. These findings shed light on key biological processes and signaling pathways involved in AML HL (**Figure 3**, **Table 4**).

**Table 4:** KEGG gene sets correlated with HL.

NAME	ES	NES	NOM p-val
**KEGG_cytokine_cytokine_receptor_interaction**	-0.5	-1.95	0
**KEGG_ECM_receptor_interaction**	-0.56	-1.94	0
KEGG_hematopoietic_cell_lineage	-0.54	-1.88	0
KEGG_cell_cycle	-0.5	-1.8	0
KEGG_dna_replication	-0.58	-1.77	0.001
KEGG_oxidative_phosphorylation	-0.48	-1.72	0
KEGG_antigen_processing_and_presentation	-0.49	-1.7	0
KEGG_Ribosome	-0.48	-1.7	0
**KEGG_Cell_Adhesion_Molecules**	-0.46	-1.69	0
KEGG_p53_signaling_pathway	-0.49	-1.63	0.002
KEGG_complement_and_coagulation_cascades	-0.47	-1.59	0.004
KEGG_base_excision_repair	-0.53	-1.58	0.016
KEGG_chemokine_signaling_pathway	-0.41	-1.57	0.001
KEGG_focal_adhesion	-0.41	-1.56	0
KEGG_Toll_Like_Receptor_Signaling_Pathway	-0.43	-1.49	0.011
KEGG_lysosome	-0.41	-1.48	0.008
KEGG_TGF_beta_signaling_pathwaY	-0.42	-1.46	0.014
KEGG_Pathways_In_Cancer	-0.35	-1.35	0.004
KEGG_Natural_Killer_Cell_Mediated_Cytotoxicity	-0.37	-1.34	0.043

### Potential molecular targeted drugs for HL using CELLMINER

We employed the CELLMINER platform to identify potential molecular targeted drugs for differentially expressed genes associated with HL. By analyzing both gene expression profiles and drug sensitivity data derived from NCI-60 cell lines, we identified a comprehensive list of differentially expressed genes linked to HL. From this analysis, several molecular targeted drugs have been identified as potential therapeutic agents for treating HL in AML patients. These include BCL2 inhibitors, histone deacetylase inhibitors, mTOR inhibitors, and other promising compounds known to target specific cellular pathways involved in disease progression. The utilization of these molecular targeted drugs alone or in combination may hold promise for effectively addressing the challenges posed by HL. The development of personalized treatment strategies based on these findings may lead to improved clinical outcomes and enhanced patient care (**Table 5**).

**Table 5:** Potential Molecular Targeted Drugs for HL.

Name	Mechanism °	FDA status	P-value
Obatoclax	Apo|BCL2	Clinical trial	0.001
Mocetinostat	HDAC	Clinical trial	0.006
Sabutoclax	MCL1	Clinical trial	0.001
CC-223	PK: MTOR	Clinical trial	0.008
Pp-242	PK: MTOR	Clinical trial	0.006
HYPOTHEMYCIN	PK: YK,MAP2K1,MAP2K2	Clinical trial	0.008
Ixazomib citrate	PSM	FDA approved	0.006
CYC-116	PK: STK,AURK	Clinical trial	0.006
Pazopanib	PK: YK,PDGFR,FGFR,KIT,VEGFR	FDA approved	0.005
6-MERCAPTOPURINE	Ds|PPAT	FDA approved	0.002
Allopurinol	AM|XDH	FDA approved	0.002
Parthenolide	Apo	FDA approved	0.001
Pazopanib	PK: YK,PDGFR,FGFR,KIT,VEGFR	FDA approved	0.000
Ixazomib	PSM	FDA approved	0.000

### The efficacy of Bcl-2 inhibitor venetoclax in the treatment of acute myeloid leukemia with HL

**Figure 4:**
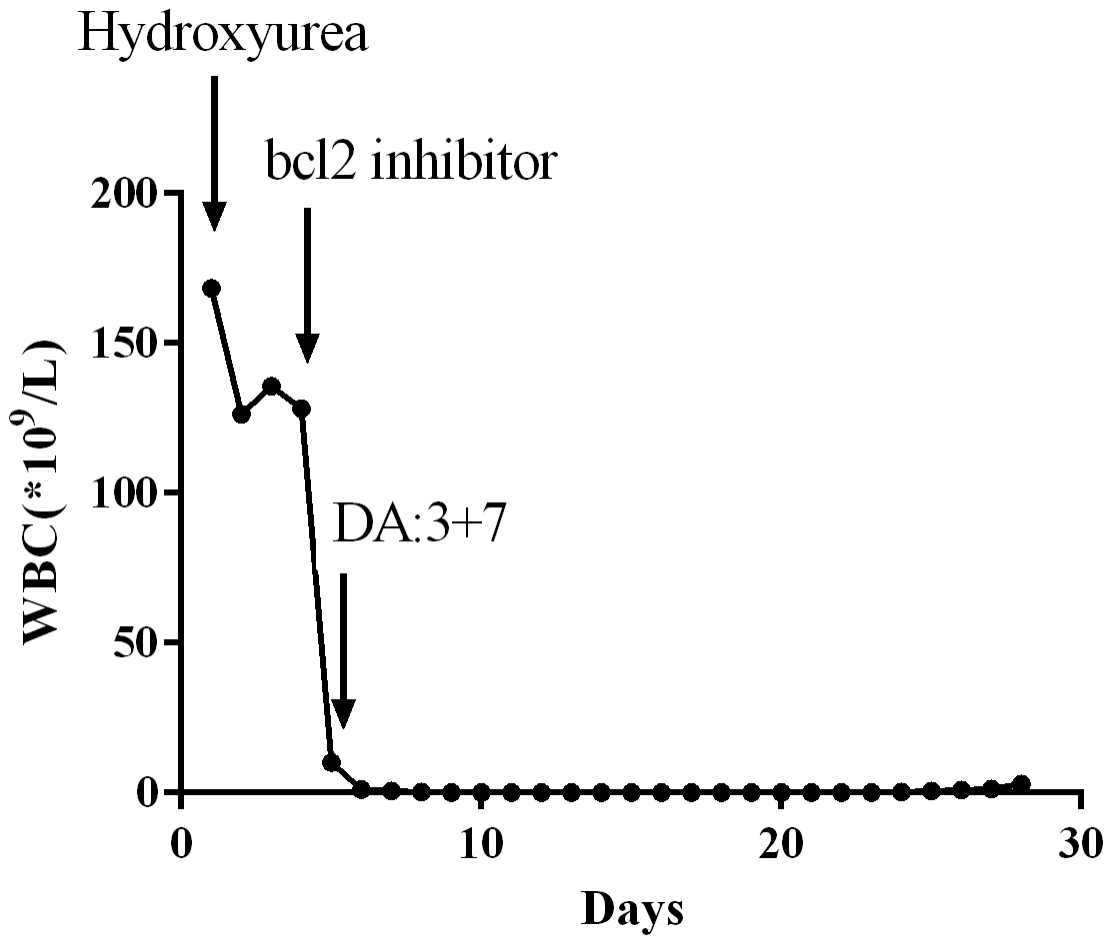
Clinical Application of Molecular Targeted Therapy Bcl-2 Inhibitor Venetoclax for HL.

Venetoclax, a Bcl-2 inhibitor, has been extensively utilized for the treatment of elderly patients diagnosed with AML, demonstrating significant therapeutic effects. In our clinical practice, we have observed that venetoclax also exhibits remarkable efficacy in AML patients presenting with HL. A 54-year-old male patient presented with symptoms such as fever and easy bruising. Laboratory tests revealed an elevated WBC count of 164*10^9^/L along with blasts observed in the peripheral blood smear. Further diagnostic investigations including flowcytometry, bone marrow cytology examination, molecular biology examination, and cytogenetic examination were conducted to confirm the diagnosis of AML with FAB Subtype: M5 Acute Monocytic Leukemia. Initially, treatment involved oral administration of hydroxyurea at a dose of 1.0 q6h to reduce WBC count. Although there was a slight decrease in WBC according to daily blood tests, the count remained above 100*10^9^/L. On the fourth day, venetoclax was initiated as part of the treatment regimen at a dosage of venetoclax 100mg once daily. Remarkably, within one day after initiating venetoclax therapy, normalization of WBC levels was achieved. Subsequently, combination chemotherapy comprising daunorubicin and cytarabine (3 + 7 regimen) was administered while continuing therapy using venetoclax. During subsequent evaluation for treatment efficacy stages later on during the course, complete remission was achieved by significantly improving the patient’s condition. Throughout this entire course of treatment, no occurrences such as acute respiratory distress syndrome (ARDS), tumor lysis syndrome (TLS), or disseminated intravascular coagulation (DIC) were observed. Venetoclax, as a Bcl-2 inhibitor, has proven to be highly effective in treating HL by rapidly reducing WBC count and decreasing the tumor burden in leukemia patients. Moreover, it lowers the risk of complications associated with HL such as leukostasis, DIC, and TLS. Venetoclax has gained widespread clinical application due to its excellent clinical efficacy (**Figure 4**).

## 4 Discussion

HL, characterized by abnormally high leukocyte counts, may represent an independent subtype of leukemia. In the context of AML, elevated leukocyte counts have been associated with a poor prognosis and increased risk of early mortality. It is believed that unique molecular biological mechanisms contribute to relapse and death in patients with high leukocyte counts (6, 12).While some studies propose considering HL as an independent subtype within AML, consensus among researchers has not yet been reached regarding this concept. Nonetheless, it is widely recognized that HL serves as a significant poor prognostic factor in AML patients. Higher levels of leukocytes are consistently associated with worse outcomes (4–6, 10, 12, 13).

In this study, we utilized X-tile software to determine the ideal threshold for WBC count in adult patients diagnosed with AML with HL. Lower leukocyte counts were associated with more favorable prognoses, while higher leukocyte counts indicated poorer prognoses in AML patients. This information can assist clinicians in assessing disease severity and making informed decisions regarding treatment strategies.

Traditionally, a threshold of 100 ×10^9^/L has been used to identify patients at super high risk or requiring urgent further investigation. However, our research suggests that lowering this threshold to 75*10^9^/L can still effectively capture individuals who may benefit from timely intervention or closer monitoring. This adjustment in the diagnostic criteria may have significant implications in clinical practice, as it enables the identification and subsequent treatment of a greater number of super high-risk patients.

Furthermore, our analysis revealed that HL showed significant associations with total WBC count, peripheral Blast ratio, bone marrow Blast ratio, and specific genetic markers such as NPM1, FLT3, DNMT3A, BCR-ABL1, HECW1, and GATA2. Patients with HL exhibited a higher number of leukemic cells in the bone marrow and peripheral blood compared to non-HL patients. Additionally, FLT3 gene mutations were present in over half of HL patients, while DNMT3A and NPM1 gene mutations were present in nearly half of the cases.

The correlation between HL and gene mutations, particularly FLT3 and DNMT3A, indicates the potential importance of these mutations in the development and progression of HL in AML. Further research is needed to elucidate the underlying mechanisms and explore targeted therapies for patients with these specific genetic markers.

Prompt and effective intervention is crucial in managing HL, as it is a critical hematological emergency associated with high-risk leukemia. Our analysis suggests that early complete remission therapy can reduce the risk of HL and improve overall survival in patients. Achieving complete remission through aggressive treatment significantly decreases the risk of HL. As patient survival time increases, the risk of HL on prognosis is completely eliminated for patients with a survival time exceeding 6 months and one year.

In pediatric patients, the optimal threshold for WBC count to predict outcomes differs, with a threshold of 165*10^9^/L identified. By striving for deeper disease remission and closely monitoring MRD levels, clinicians can potentially eliminate the risk of HL relapse, thereby improving the overall prognosis for these patients. This underscores the need for personalized treatment strategies that aim to achieve MRD negativity in order to optimize outcomes.

Differential gene analysis identified several genes that are differentially expressed between HL and non-HL patients, including cell adhesion molecules and cytokines. Dysregulation of these genes may contribute to abnormal cell adhesion, migration, and immune responses, which could impact the development and progression of HL.

According to the differential gene analysis conducted on the TCGA database, valuable insights into HL and its association with cell adhesion and cytokines have been provided. To further understand the molecular processes of HL in acute myeloid leukemia (AML), METASCAPE analysis and gene set enrichment analysis (GSEA) were performed. These analyses revealed several gene sets negatively correlated with HL, including cytokine-cytokine receptor interaction, extracellular matrix-receptor interaction, cell cycle regulation, DNA replication, cell adhesion molecules, cytokine signaling pathways, adhesion signaling pathways, and epithelial-mesenchymal transition (EMT). These findings provide insights into the key biological processes and signaling pathways involved in AML with HL.

Furthermore, potential molecular targeted drugs for differentially expressed genes associated with HL were identified using the CELLMINER platform. Analysis revealed promising drugs, including BCL2 inhibitors, histone deacetylase inhibitors, which target specific cellular pathways involved in disease progression. The use of these molecular targeted drugs, either as monotherapy or in combination, may hold promise in effectively addressing the challenges posed by HL and improving clinical outcomes.

One promising drug is venetoclax, a Bcl-2 inhibitor, which has shown significant therapeutic effects in AML treatment, particularly in elderly patients. In clinical practice, venetoclax has also demonstrated significant efficacy in AML patients with HL.

Recent studies (14–16) have shown promising results with the combination therapy of venetoclax and other targeted drugs. For example, the combination of venetoclax, azacitidine, and daunorubicin achieved a higher complete remission rate in newly diagnosed acute monocytic leukemia patients. Additionally, the combination of venetoclax and azacitidine showed efficacy in patients with FLT3-ITD mutations. The combination therapy of Venetoclax and Azacitidine, along with Chidamide, has been used for the treatment of newly diagnosed acute monocytic leukemia patients, achieving a complete remission rate as high as 92.3%. For patients with FLT3-ITD mutations, the complete remission rate reaches 100%, while for those with TP53 mutations, it is 66.7%. According to the 2022 European LeukemiaNet (ELN) guidelines classification, the rates of CRc (complete remission with incomplete hematologic recovery) among low-, intermediate-, and high-risk patients are 100%, 100%, and 80% respectively.

In conclusion, our study provides valuable insights into the ideal threshold for WBC count in AML patients with HL, the association between HL and specific genetic markers, and the impact of early high-quality disease remission on prognosis. These findings have important implications for dynamic assessment of hyperleukocytosis risk, treatment decisions, and overall survival outcomes in AML patients with HL.

## Data availability statement

The original contributions presented in the study are included in the article/supplementary material. Further inquiries can be directed to the corresponding author.

## Ethics statement

This study was approved by the Human Research Ethics Committee of First Affiliated Hospital of Xinxiang Medical University (NO.2020156). The studies were conducted in accordance with the local legislation and institutional requirements. Written informed consent for participation in this study was provided by the participants’ legal guardians/next of kin.

## Author contributions

GL: Conceptualization, Data curation, Formal analysis, Funding acquisition, Investigation, Methodology, Project administration, Resources, Software, Supervision, Validation, Visualization, Writing – original draft, Writing – review & editing. WL: Data curation, Formal analysis, Investigation, Methodology, Resources, Validation, Visualization, Writing – original draft.
